# Predictable dental rehabilitation in maxillomandibular reconstruction with free flaps. The role of implant guided surgery

**DOI:** 10.4317/medoral.19116

**Published:** 2014-08-17

**Authors:** José L. Cebrian-Carretero, Jorge Guiñales-Díaz de Cevallos, José A. Sobrino, Tomás Yu, Miguel Burgueño-García

**Affiliations:** 1 MD, DDS. Faculty, Oral and Maxillofacial Surgery Department, University Hospital La Paz, Madrid, Spain; 2 MD. Trainee, Oral and Maxillofacial Surgery Department, University Hospital La Paz, Madrid, Spain; 3 MD, DDS. Private practice; 4 MD. Head of Department, Oral and Maxillofacial Surgery Department, University Hospital La Paz, Madrid, Spain

## Abstract

The reconstruction of maxillomandibular defects secondary to oral cancer surgery, represent a great challenge for Maxillofacial surgeons. During the last decades the reconstructive surgery has experimented a big advance due to the development of the microsurgical techniques. At present, we are able to reconstruct complex defects using free flaps that provide both soft and bone tissue. Fibula, iliac crest and scapula free flaps have been the three classic options for the maxillomandibular reconstruction owing to the amount of bone that this flaps provide, allowing the posterior dental rehabilitation with implants. Today, our objective it is not only the aesthetic reconstruction, but also the functional reconstruction of the patients enhancing their life quality. Guided implant surgery in free flap reconstructed patients has become an essential tool, helping to define the exact position of the dental implant in the flap. In this way it is possible to look for the areas with better bone conditions, avoiding the osteosynthesis material used to fixate the flap with the native bone and deciding the best biomechanical option, in terms of number and situation of the implants, for the future dental prostheses. In summary, using the guided implant surgery, it is possible to design an exact and predictable dental implant rehabilitation in patients with oral cancer who are reconstructed with free microvascular flap, resulting in an optimal aesthetic and functional result.

** Key words:**Oral cancer, mandibulectomy, maxillectomy, microvascular reconstruction, fibula flap, dental implant, guided surgery.

## Introduction

During the second half of the 20th century, a great revolution in the surgical treatment of oral cavity tumours took place, evolving from a destructive mentality, in which just the tumour resection was performed without considering the normalization of the affected functions, to a reconstructive mentality in which the functional and aesthetic properties were restored. Reconstruction plates, free grafts, local and pediculated flaps and finally free microvascular flaps have been used progressively in this reconstructive aim. While the initial goal of reconstructive surgery was to cover de created defect, it was from the last decades of the 20th century, when it began to arise the current purpose of replacing each tissue with one of similar characteristics (1-4).

In the field of bone reconstruction of the facial skeleton, we must take into account that both the maxilla and the mandible have the peculiarity of being carriers of the dental arches and consequently the occlusal function should be also considered in our reconstructions. We know that the placement of implant supported prostheses offers to these patients a definitive solution for the recovery of this function and at the same time they help to improve other features (5-7).

Microvascular iliac crest, scapula and fibula flaps have been the main options for this bone and dental rehabilitation. Fibula flap was first described by Taylor and Gilbert at the end of the 1970s, but it wasn’t until 1989 when Hidalgo employed it for mandibular reconstruction. Since then, it has become widespread thanks mainly to its versatility by offering great length of bone with a good quality pedicle (1,8).

During the last years, several articles have explored the benefits of different reconstruction flaps employed in the mandible. Some highlight the advantage of the iliac crest allowing the immediate placement of implants during the reconstructive phase, while in the fibula this should be defer. Others stand out the length of the fibula that allows complete mandibular and maxillary reconstructions. In any case, the disadvantage of both flaps is the difficulty of precise implant placement in those situations in which, after the tumour resection, the dental occlusion has been lost and the flap bone is not of the same anatomical features as the original one. In this way, many of the placed implants latter can’t be rehabilitated or the rehabilitation achieved result in a no functional dental occlusion (4,6,9).

History of implant placement in bone grafts dates back to the last quarter of the 20th century, first in free grafts (5), later in pediculated flaps (7) and finally in microvascular flaps (10-16). Currently, guided implantology surgery systems allow us to plan and place implants in a virtual manner based on a computerized tomography (CT) that duplicates the bone anatomy of the patient. A splint, supported on the tissues of the patient, is made through computer planning programs, stereolithography and rapid prototyping, and used during the surgery, indicating us the precise position of each implant so the final location will match the planned situation. Also, guided implantology surgery allow us to outline how the definitive prosthesis will be ignoring the problems of malocclusion and not rehabilitated implants occurred when we perform classic implant surgery in microvascular bone flaps (17-18).

In this paper we intend to show our experience in the use of guided surgery for implant placement in microvascular fibula graft and analyse its advantages, disadvantages and possible complications.

## Patient and Methods

We present four patients undergoing oncological resections of the maxilomandibular complex, immediate or differed reconstruction with free fibula flaps and subsequently dental rehabilitation with implant supported prostheses, using guided surgery systems.

**Figure 1:**
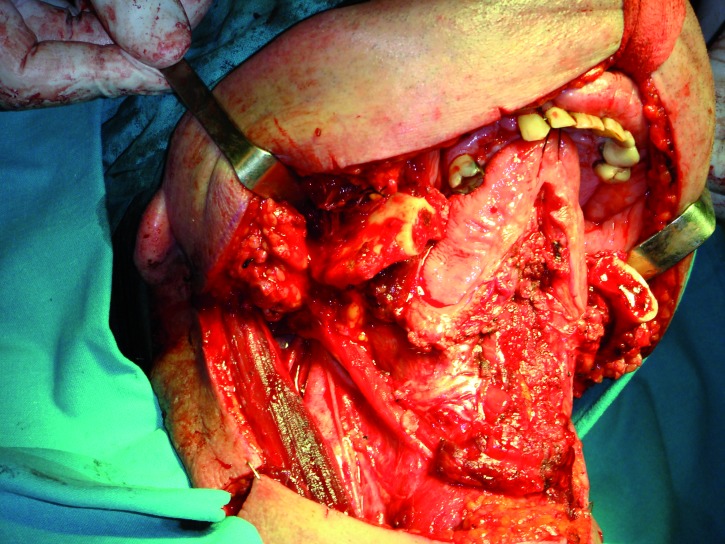
Intraoperative detail of one of the patients with an angle-to-angle mandibular resection.

**Figure 2:**
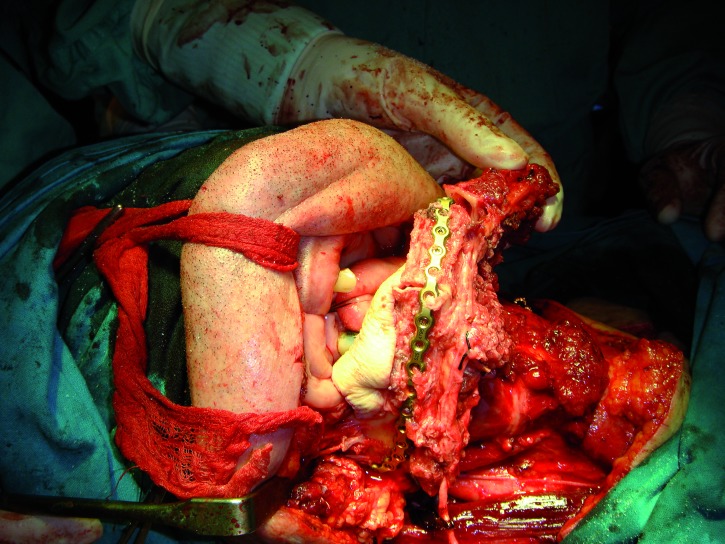
Immediate reconstruction using a microvascular fibula flap and a reconstruction plate.

**Figure 3:**
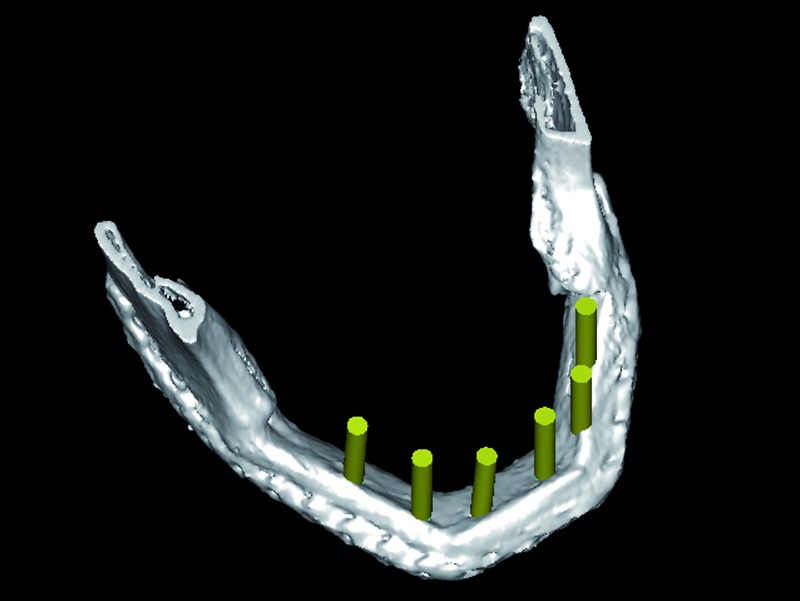
Virtual implant placement avoiding the osteosynthesis material and selecting the most favorable positions for the implants in terms of the bone quality.

**Figure 4:**
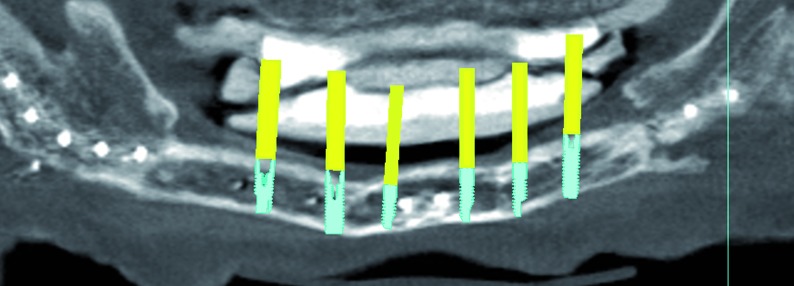
The future prosthesis design.

**Figure 5:**
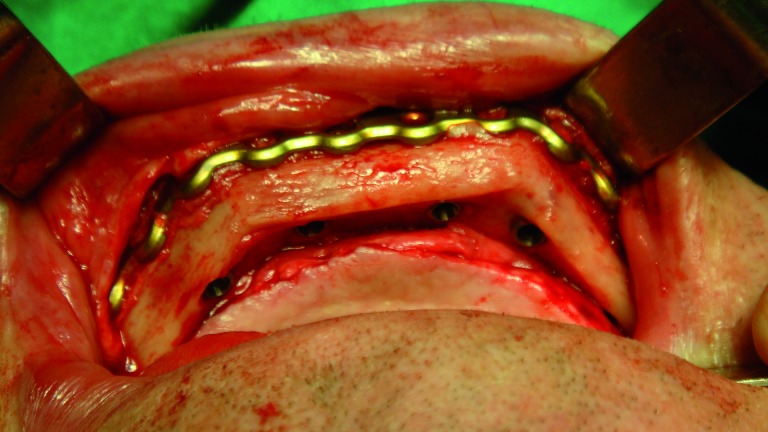
Final position of the implants.

In three cases the reconstructed bone was the mandible. All of them were men with an average age of 54 years. In two of the three patients, angle-to-angle mandibulectomy was performed and in the third case a left mandibular resection from symphysis to angle was carried out. In all three cases the histological diagnosis was squamous carcinoma of the oral cavity and all received postoperative radiation therapy. Two of these patients were immediate reconstructed while in the third one a differed reconstruction was performed after completing the radiation therapy. (Fig. 1). The flap fixation to the mandible remnant was conducted in all patients with titanium reconstruction plates which were not removed to carry out the implant treatment (Fig. 2). During the planning phase of the guided surgery, those plates-free areas were selected to place the implants (Figs. 3,4). In the three cases, it was necessary to rehabilitate the entire lower arcade. We use six implants in the two cases with angle-to-angle resections, while in the patient of hemimandibular resection four implants were placed in the fibula flap and three in the edentulous mandible remnant. In total, sixteen implants were placed (Astra Tech, Osseospeed ™) in the microvascular flaps and all of them were carried out in a period of time deferred between 6-12 months after the reconstruction (Fig. 5). With regard to the opposite arcades, the two patients of total mandibular reconstruction, who had an edentulous maxilla, were treated at the same time with six implants for the maxillary occlusal rehabilitation. The patient with the hemimandibular reconstruction held a fixed dental prosthesis in the front sector and removable prosthesis in the posterior sector.

**Figure 6:**
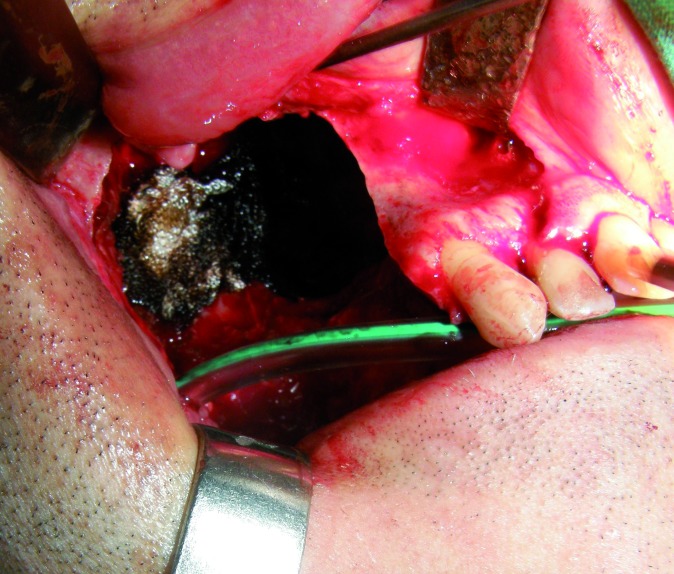
Maxillectomy defect from the canine to the pterygoid plate.

**Figure 7:**
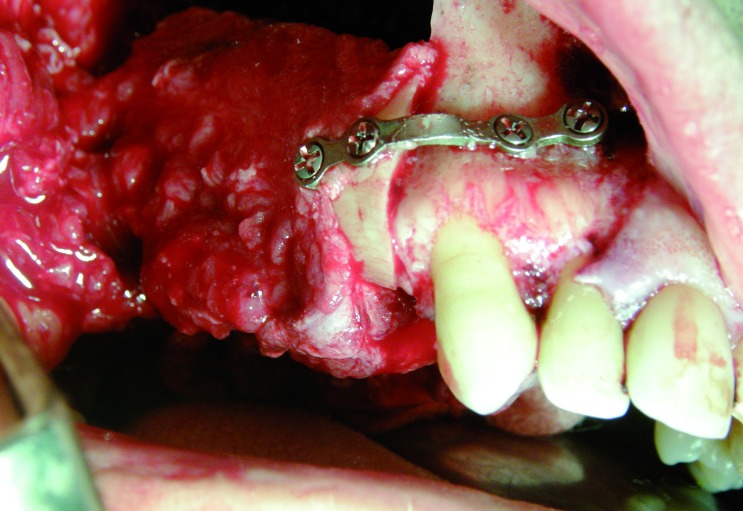
Reconstruction with fibula flap.

**Figure 8:**
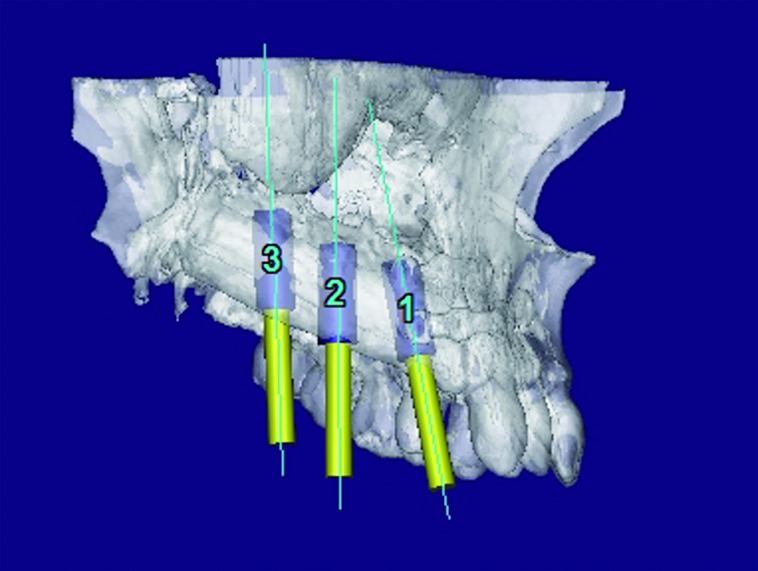
Virtual position of the implants.

The last case was a patient of 45 years affected of a maxillary odontogenic myxoma who required a segmental maxillectomy involving from canine to the pterygoid plate (Fig. 6). An immediate reconstruction with free fibula flap was realised, fixating it with titanium miniplates (Fig. 7). The patient did not receive radiotherapy and three titanium implants were placed (Astra Tech Osseospeed ™) for the dental rehabilitation in a second surgical stage (Fig. 8). In the mandibular dental arch the patient wore their natural teeth. In all the cases, the implants used had diameters of 4 mm and lengths ranging between 8 and 17 mm.

**Figure 9:**
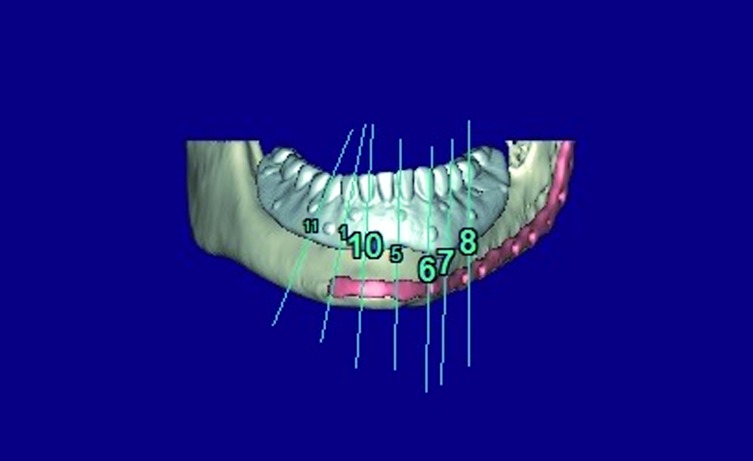
Design of a bone-supported surgery splint on the CT.

**Figure 10:**
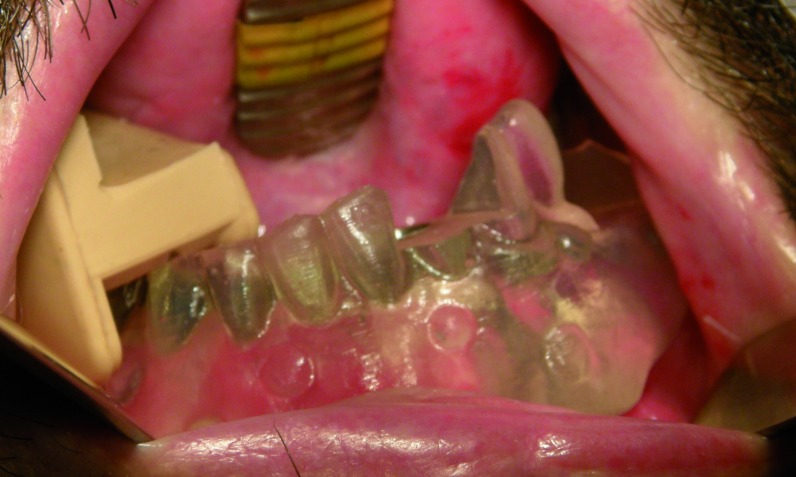
Mucosa-supported surgery splint.

Regarding to the support of the splint used for the guided surgery, it was held on the mucosa in two cases while in the third patient a bone support was selected because the patient didn’t have enough buccal vestibule to accommodate the splint after the mandible reconstruction. In the patient with a maxillary reconstruction a dental support was chosen (Figs. 9,10).

All patients received preoperative antibiotic prophylaxis with 2 grams i.v. amoxicillin-clavulanate administered 30 minutes prior to intervention. After the surgery, treatment was continued with amoxicillin-clavulanate 875 mg vo during 7-10 days. All three patients of mandibular reconstruction were operated under general anaesthesia, while in the fourth patient the surgery was carried out under local anaesthesia. An osseointegration period of 4 months was respected in all cases and the second surgical stage was performed under local anaesthesia. The healing period with transepithelial pillars was of 15 days.

## Results

**Figure 11:**
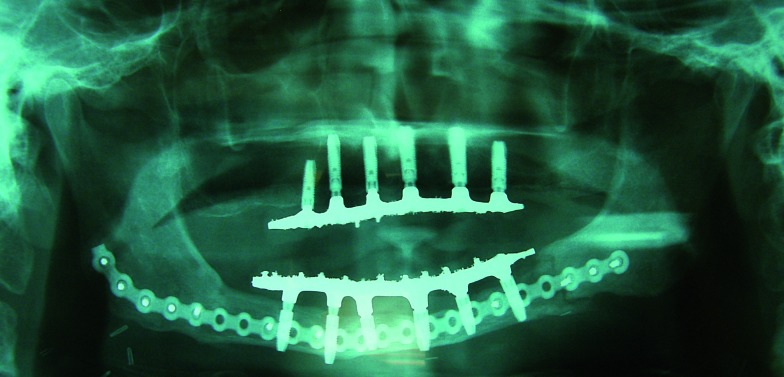
Radiological control of the position of the implant of the patient of figure 1-5. Accurate position was achieved when compared with the virtual implant placement (Figs. 3,4).

**Figure 12:**
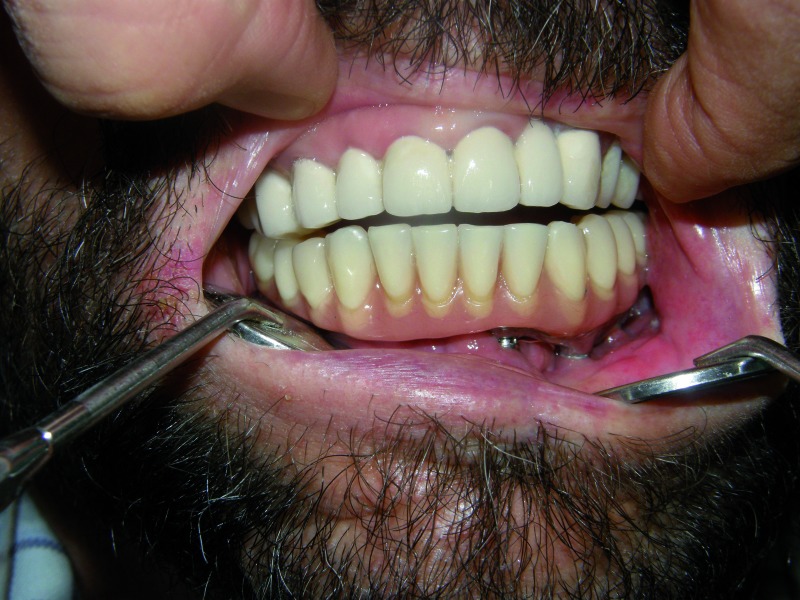
Final dental prosthesis of the patient with angle to angle mandibular resection.

The initial osseointegration was of the 100% and after 18 months of function, no implants have failed. During this period the peri-implant bone loss was less than 1 mm and all the implants could be rehabilitated. Planning and placement were precise with a margin of error of less than 1 mm, between virtual surgery and the final position in the patient (Fig. 3). Custom trays and addition silicone were used for the implant impressions. The prosthetic solution for complete mandibular rehabilitation was hybrid prosthesis with acrylic teeth and for the maxillary free end a ceramometal fixed prosthesis was made (Figs. 11,12).

Regarding to the technical complications, in the patient requiring angle to angle reconstruction it was impossible to correctly adapt a radiologic splint over the mucosa for the realization of the planning CT, so the splint for the guided surgery was bone supported, being necessary to lift a flap on the skin palette of the fibula. In this same patient the prosthetic phase was very complex because of the total lack of bucal vestibule. Other problems were raised during the insertion of the implants in this patient, due to the limited oral opening (35 mm). In this case the first five millimetres of the drilling were made using shorter drills and then the splint was removed to complete the drilling phase and the implants placement. This patient also presented a wound dehiscence which was treated conservatively.

Dental occlusion of the four patients is satisfactory and they have recovered the chewing function being able to eat a normal diet. In addition the reconstruction intended to achieve a proper tongue mobility to facilitate swallowing and an important aesthetic improvement has also been accomplish ( Table 1).

**Table 1:**
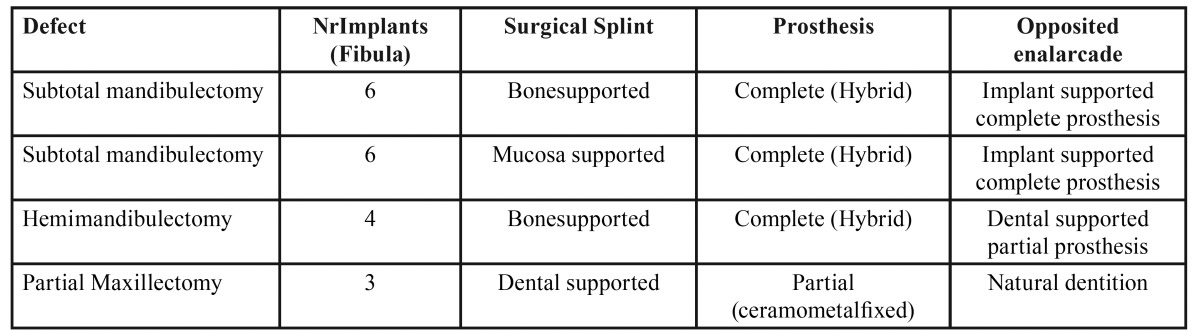
Summary of reported cases.

## Discussion

The development of facial reconstructive surgery during the last decades has allowed, not only saving the life of many oncologic patients, but also improving their quality of life. The ability to eat and carry a normal diet are directly dependent on the good chewing function. Microvascular flaps employment and the progress of implantology have largely helped to achieve these goals (4,8).

With regard to the employed flaps, fibula and iliac crest remains as the most versatile options, although it is necessary to individualize each case to perform a customized reconstruction for every single patient. The major advantages of the fibula microvascular flap for the maxillomandibular reconstruction and especially for dental rehabilitation are: its length, which allows using up to 25 cm of bone for the reconstruction and the chance of shaping it, the possibility of performing multiple osteotomies given its extraordinary periosteal vascularization, and the minimum morbidity in the donor area. Consequently its principal indications would be large mandibular reconstructions associated with important soft tissue intraoral defects, reconstruction of partial or complete defects involving the mandibular symphysis and the reconstructions of mandibular posterior sector including ramus and condyle (1,8,19). Its disadvantage is the low height of the fibula bone hindering the functional rehabilitation with osseointegrated implants (20) that also should be placed essentially in a deferred manner because the incompatibility with the osteosynthesis material required to fix the osteotomies6. In any case, implant rehabilitation would be possible whenever we have at least 10 mm of bone height and 5 mm in width (20,21).

The main problem referred to the implantology in microvascular flaps, has been the accuracy in placing implants to achieve a correct emergency of the prosthetic devices and ultimately a good occlusion. In this sense, it is not uncommon that many of the placed implants may not be rehabilitated later. Guided implantology surgery systems have overcome many of these problems. Virtual planning allows us to.

1. Place the implants there where the radiological quality of bone is optimal by precisely adjusting the length and width of the implants.

2. Planning the emergence of the prosthetic devices so that placed implants could be rehabilitated.

3. Avoid the necessity of removing the osteosynthesis material by placing the implants, if possible, where there are no screws.

4. To perform minimally invasive surgery without deperiostizating the grafted bone.

5. Planning immediate prosthesis that can be placed in the postoperative period to improve the function and adaptation of soft tissues (12,18,22-24).

To perform guided surgery it is necessary.

1. An image test, CT scan, allowing us to analyze accurately the maxillary bone anatomy and carry out the virtual surgery.

2. A planning computer program, which in our case has been the tool Facilitate ™ for the SimPlant ™ program of Materialise Dental ™.

3. A stereolithographic cast that translates the planning information from the CT to the patient (22,25).

In this sequence of requirements we must consider that.

1. Before referring the patient to the X-ray consultation, we need to decide the type of support for the surgical splint. If we are going to perform a surgery without flap, which is the ideal way, the patient must wear a barium splint while the CT is performed. To be useful, the cast must conform accurately to the mucosa of the patient and stay stable so you we can infer the thickness of the mucosa and adjust the planning considering the ideal implants dimensions. This tends to be difficult in those cases of complete mandibular reconstruction using fibula flaps, especially if there is an intraoral soft tissue defect and a skin patch has been designed. In those cases in which it is impossible to adapt the radiological splint to the mucosa, the surgical splint will be bone or dental supported. We must bear in mind that both bone and mucosal supported splints should be fixed using specific screws in order to avoid any displacement that could affect to the treatment precision (26-28).

2. It is necessary to control the planning programme so we can be able to make use of all its advantages.

3. Finally, we must take into account the height of the splint and the space between this one and the bone so we must use larger drills, usually between 18 and 25 mm. This can complicate the surgery especially in the mandibular region, and even more if the patient does not have a good oral opening. In fact, in one of our patients we could not use the planned drills and standard ones were used to prepare the first 5 mm of the drilling and then remove the splint to complete the surgery. Fortunately it was a bone supported splint and we were able to control the depth of insertion, but in cases dental or mucosal support, this may represent a problem (22-24,27,28).

In any case the concordance between planned and final surgery in cases in which we attend all of these premises is very high, with differences smaller than 1 mm, allowing, in our cases, to rehabilitate all the implants and achieve acceptable occlusions even in complex cases. In addition, thanks to the stability of the implant supported prostheses, the complications derived from soft tissue, could be resolved without complex retouching of the flap or the mucosa.

Regarding the prognosis of these implants, there are authors who support that the implants placed in microvascular bone flaps have demonstrated to have better prognosis when compared to classic ones, with statistically differences, thanks to their the great vascularization of this flaps. In this sense, we have observed in our patients that the peri-implant bone resorption was similar to the accepted as normal in conventional implantology (13).

Finally, the prosthetic solution must be individualised in each case according to the number of implants that can be placed, the biomechanic situation of the masticatory system, the dentition of the antagonistic arch, sensory deficits of the patient and their oral hygiene. In free ends we will prefer fixed ceramometal prostheses, while in complete reconstructions we must consider that fixed prostheses require a larger number of implants, a more complex occlusal adjustment and exhaustive hygiene. This may provide greater satisfaction to the patient even though it is a more expensive treatment.

## Conclusions

In conclusion, we must say that while healing remains our priority, the development of osseointegrated implantology and micro-surgery techniques has ostensibly improved the integral treatment of oncologic patients. Nowadays, we have the possibility of offering to our mandibulectomized patients a microvascular reconstruction with fibula flap and a dental rehabilitation with implant supported or implant retained prostheses that will enhance their facial harmony and their quality of life. This treatment gives answer to the major demands of these patients after surgical and radiotherapy treatment, showing a high satisfaction index in the different surveys.
